# Gene Expression Profile Reveals a Prognostic Signature of Non–MSI-H/pMMR Colorectal Cancer

**DOI:** 10.3389/fcell.2022.790214

**Published:** 2022-02-17

**Authors:** Zaoqu Liu, Hui Xu, Xiaoyong Ge, Siyuan Weng, Qin Dang, Xinwei Han

**Affiliations:** ^1^ Department of Interventional Radiology, The First Affiliated Hospital of Zhengzhou University, Zhengzhou, China; ^2^ Interventional Institute of Zhengzhou University, Zhengzhou, China; ^3^ Interventional Treatment and Clinical Research Center of Henan Province, Zhengzhou, China; ^4^ Department of Colorectal Surgery, The First Affiliated Hospital of Zhengzhou University, Zhengzhou, China

**Keywords:** microsatellite-stability, colorectal cancer, gene signature, recurrence, prognosis

## Abstract

Studies have demonstrated that non–MSI-H/pMMR colorectal cancer (CRC) has a worse prognosis and relapse rate than microsatellite instability-high (MSI-H)/mismatch repair deficient (dMMR) CRC. Hence, searching for a novel tool to advance the prognostic management of non–MSI-H/pMMR CRC is vital. In this study, using three independent public cohorts and a clinical in-house cohort, we developed and validated a microsatellite stable–associated signature (MSSAS). The initial signature establishment was performed in GSE39582 (*n* = 454). This was followed by independent validation of this signature in The Cancer Genome Atlas–CRC (*n* = 312), GSE39084 (*n* = 54), and in-house cohort (*n* = 146). As a result, MSSAS was proven to be an independent risk factor for overall survival and relapse-free survival in non–MSI-H/pMMR CRC. Receiver operating characteristic analysis showed that MSSAS had a stable and accurate performance in all cohorts for 1, 3, and 5 years, respectively. Further analysis suggested that MSSAS performed better than age, gender, and the T, N, M, and AJCC stages, adjuvant chemotherapy, tumor mutation burden, neoantigen, and *TP*53, *KRAS*, *BRAF*, and *PIK3CA* mutations. The clinical validation was executed to further ensure the robustness and clinical feasibility of this signature. In conclusion, MSSAS might be a robust and promising biomarker for advancing clinical management of non–MSI-H/pMMR CRC.

## Introduction

Despite tremendous advances in the treatment of cancer, colorectal cancer (CRC) remains the second leading cause of cancer-related deaths (Carethers and Doubeni, 2020). Based on mutational status, CRC can be divided into mismatch repair deficient (dMMR)/microsatellite instability-high (MSI-H) and mismatch repair proficient (pMMR)/non–MSI-H tumors (Ganesh et al., 2019; Endo et al., 2020). Chemotherapy alone (5-fluorouracil) or in combination with radiation is the standard treatment for patients with advanced CRC (Maiuthed et al., 2018; Dekker et al., 2019). Although non–MSI-H/pMMR CRC has better efficacy for 5-fluorouracil–based chemotherapy, non–MSI-H/pMMR CRC patients have demonstrated a worse prognosis and higher relapse rate (Lizardo et al., 2020). In recent years, immunotherapy has attracted tremendous attention due to its significant effect on solid tumors, including CRC (Liu et al., 2021a). Accumulating evidence has revealed that immunotherapy improves the prognosis of MSI-H/pMMR CRC patients but has no effect on non–MSI-H/pMMR CRC (Biller and Schrag, 2021). Besides, non–MSI-H/pMMR CRC patients are likely to develop resistance after immunotherapy (Kather et al., 2018). Considering the dismal prognosis, recurrence, and the unsatisfactory immunotherapeutic outcomes of non–MSI-H/pMMR CRC, assessing the risk of non–MSI-H/pMMR CRC for further clinical intervention is warranted.

We hypothesized that comprehensively identifying core prognosis–related genes to construct a predictive model would enhance the accuracy of prognosis evaluation for non–MSI-H/pMMR CRC patients. Commonly used genetic testing techniques such as quantitative real-time polymerase chain reaction (qRT-PCR) are currently defective in detecting a large number of genes (Liu et al., 2021b), while the development of bioinformatics has made this possible. Presently, it is very easy to use large-scale genes for subsequent analyses. Furthermore, with the tremendous progress of machine learning, such as the least absolute shrinkage and selection operator (LASSO) algorithm, the most important elements in gene expression profiles can be determined and models with powerful extrapolation capabilities fitted (Liu et al., 2021c).

In our study, three independent public cohorts (*n* = 820) were used to develop and validate a microsatellite stable–associated signature (MSSAS). A total of 146 non–MSI-H CRC tissues were collected for external verification to assess the robustness and accuracy of MSSAS. Finally, a novel six-genes signature was reported. This signature not only offers robust and high reliability in identifying patients at high recurrence risk and worse overall survival but also can be readily applied into clinical practice due to the inexpensiveness and simplicity of the PCR-based assays. Taken together, MSSAS provides an excellent platform for assessing prognosis and recurrence risk and might be a promising tool to facilitate the clinical management of non–MSI-H/pMMR CRC patients.

## Materials and Methods

### Data Collection and Processing

**FIGURE 1:**
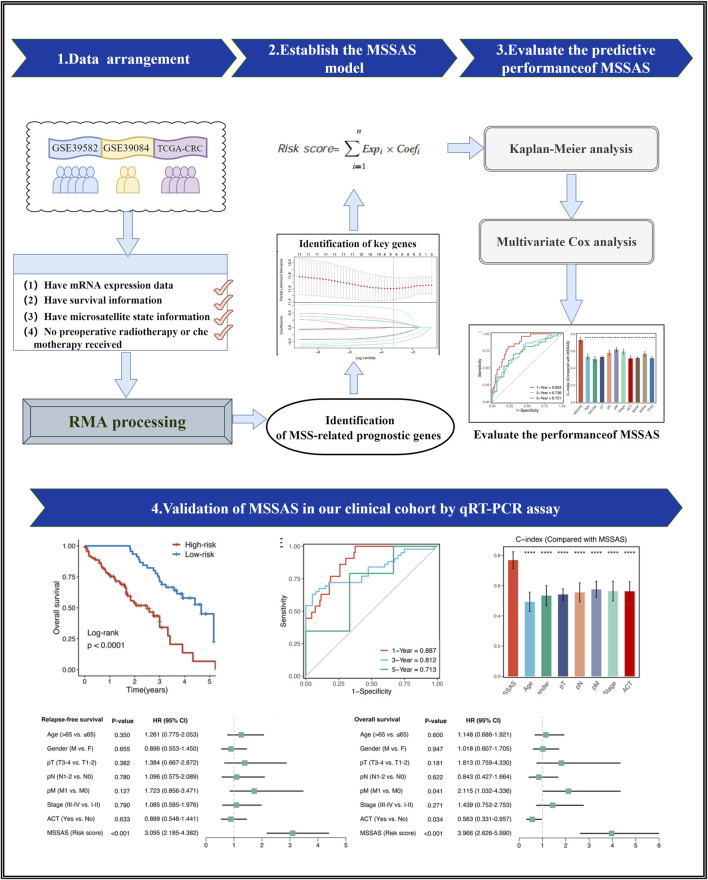
The flowchart of this study.

The overall flowchart is shown in Figure 1. Three independent CRC cohorts were retrieved from the Gene Expression Omnibus (http://www.ncbi.nlm.nih.gov/geo) and The Cancer Genome Atlas (TCGA, https://portal.gdc.cancer.gov), including GSE39582, GSE92921, and TCGA-CRC. The microarray data were normalized by the robust multi-array average algorithm, and the RNA-seq raw count data were performed by transcripts per kilobase million and log-2 transformation. A combat algorithm was used to remove the batch effects in Meta-Cohort (derived from three microarray data sets). In these three cohorts, patients who met the following criteria were retained: those with 1) mRNA expression data, 2) survival information, 3) microsatellite state information, and 4) no preoperative radiotherapy or chemotherapy received. Ultimately, TCGA-CRC had 54 MSI-H CRC and 312 non–MSI-H CRC; GSE39582 had 77 dMMR CRC and 459 proficient mismatch repair (pMMR) CRC; and GSE39084 had 16 MSI-H CRC and 54 non–MSI-H CRC. The detailed baseline is summarized in Supplementary Table S1.

### Identification of Microsatellite Stable–Related Prognostic Genes

To identify the microsatellite stable (MSS)–related prognostic genes, we developed a pipeline as follows: 1) across the three cohorts, the *limma* package was applied to perform differential analysis between the non–MSI-H/PMMR CRC and MSI-H/dMMR CRC groups with an FDR < 0.01; the overlap of the three cohorts was defined as MSS-related genes. Subsequently, univariate Cox regression analysis was performed on these genes. Ultimately, genes with a hazards ratio (HR) consistently >1 or <1 and *p* < 0.05 across the three cohorts were considered as stable prognostic genes.

### Establishment of Microsatellite Stable–Associate Signature

The LASSO is a prevalent machine learning algorithm, which was employed for variable selection to identify key genes in this study. By 10-fold cross-validation, the optimal lambda was generated when the partial likelihood deviance reached the minimum value. To make our signature more practical, we further adopted the stepwise Cox algorithm and generated a risk score formula which includes the regression coefficients of weighted genes.

### Human Colorectal Cancer Specimens and Quantitative Real-Time Polymerase Chain Reaction Analysis

A total of 146 frozen resected CRC tissues with non–MSI-H were collected from The First Affiliated Hospital of Zhengzhou University. The clinical staging of the specimens was based on NCCN (2019) guidelines; see Supplementary Table S1 for the detailed baseline data of our samples. Total RNA was isolated with RNAiso Plus reagent (Waltham, MA, United States) as described previously (Liu et al., 2021b; Liu et al., 2021c; Liu et al., 2021d; Zhang et al., 2021). The primer sequences of the included six genes and *GAPDH* are shown in Supplementary Table S2. The details of the qRT-PCR analysis are described in the Supplementary Material.

### Statistical Analysis

The *survival* package was used to conduct Kaplan–Meier and multivariate Cox regression analyses. The optimal cut-off value was determined by the *survminer* package. The *timeROC* package was performed to plot time-dependent receiver operating characteristic (ROC) curves, and comparisons of the concordance index (C-index) among the variables were conducted by using the *compareC* package. All data processing, statistical analyses, and plotting were conducted in R 4.0.5 software.

## Results

### Construction and Validation of Microsatellite Stable–Associated Signature

**FIGURE 2:**
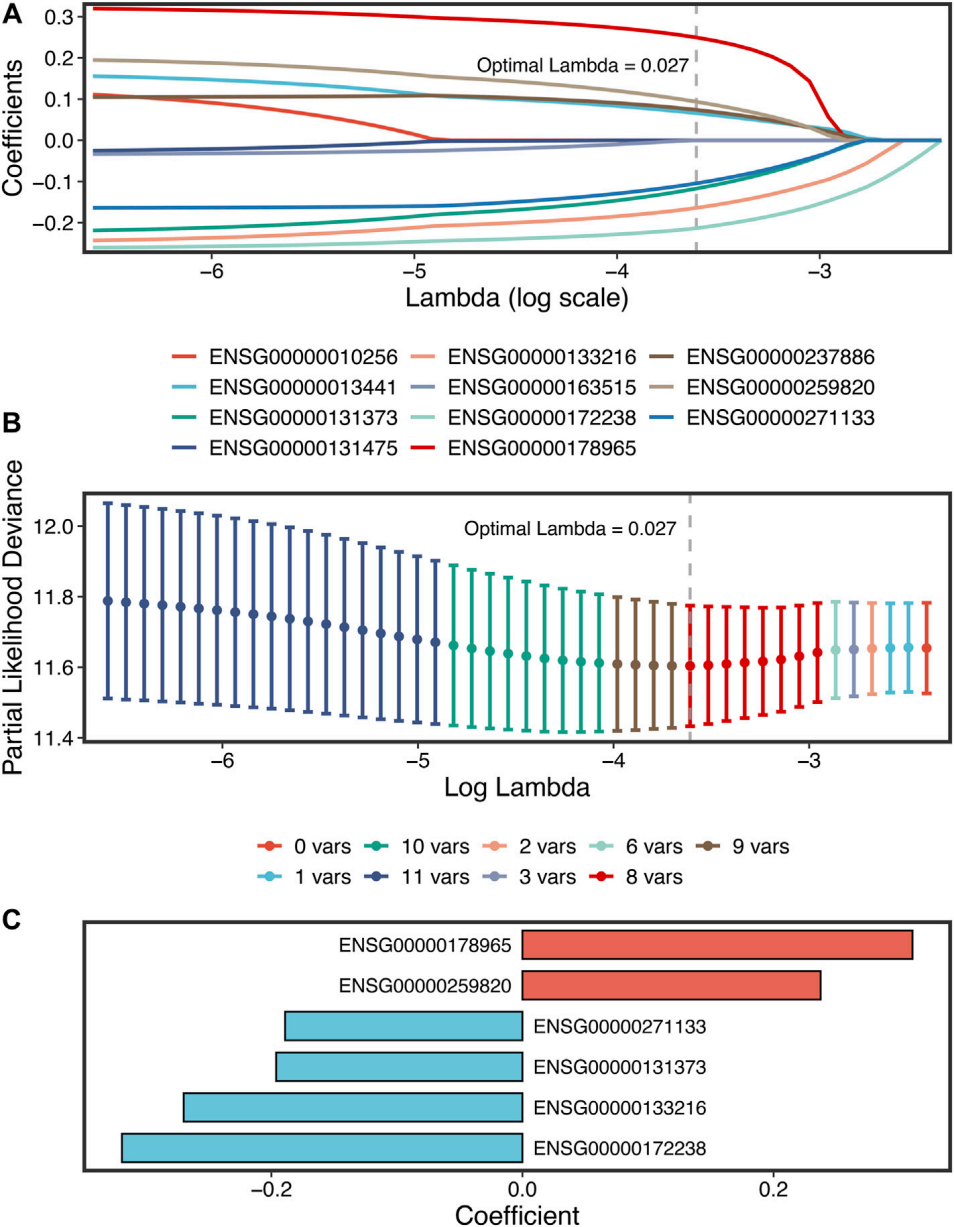
The development of the MSSAS signature based on the LASSO stepwise Cox regression algorithms. **(A)** Ten-fold cross-validations to tune the parameter selection in the LASSO model. **(B)** LASSO coefficient profiles of the candidate genes for MSSAS construction. The vertical lines are drawn at the optimal values by minimum criteria. **(C)** Coefficients of six genes finally obtained in stepwise Cox regression.

**FIGURE 3:**
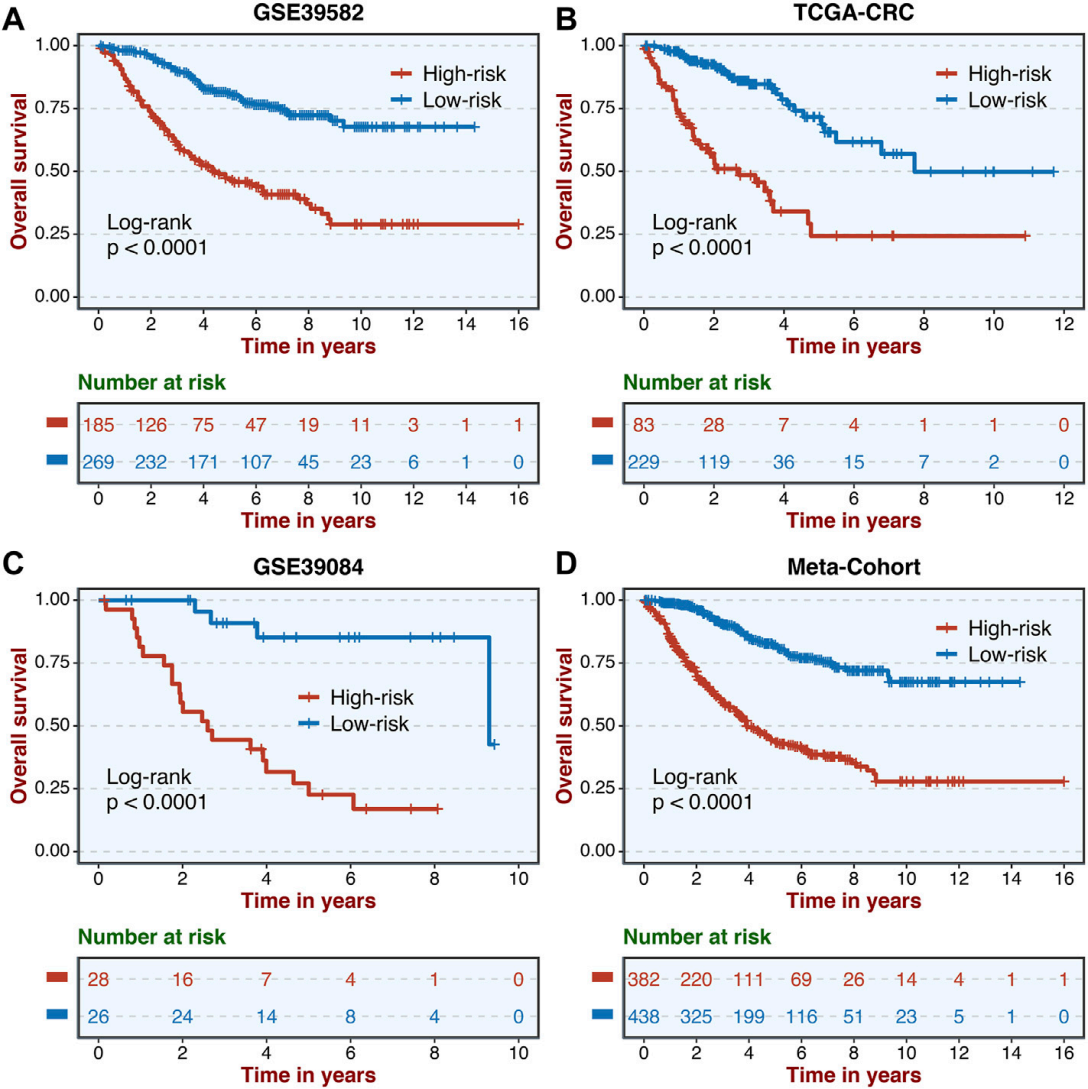
Kaplan–Meier analysis of OS according to MSSAS. Kaplan–Meier curves of OS according to the MSSAS in GSE39582 **(A)**, TCGA–CRC **(B)**, GSE39084 **(C)**, and Meta-Cohort **(D)**.

**FIGURE 4:**
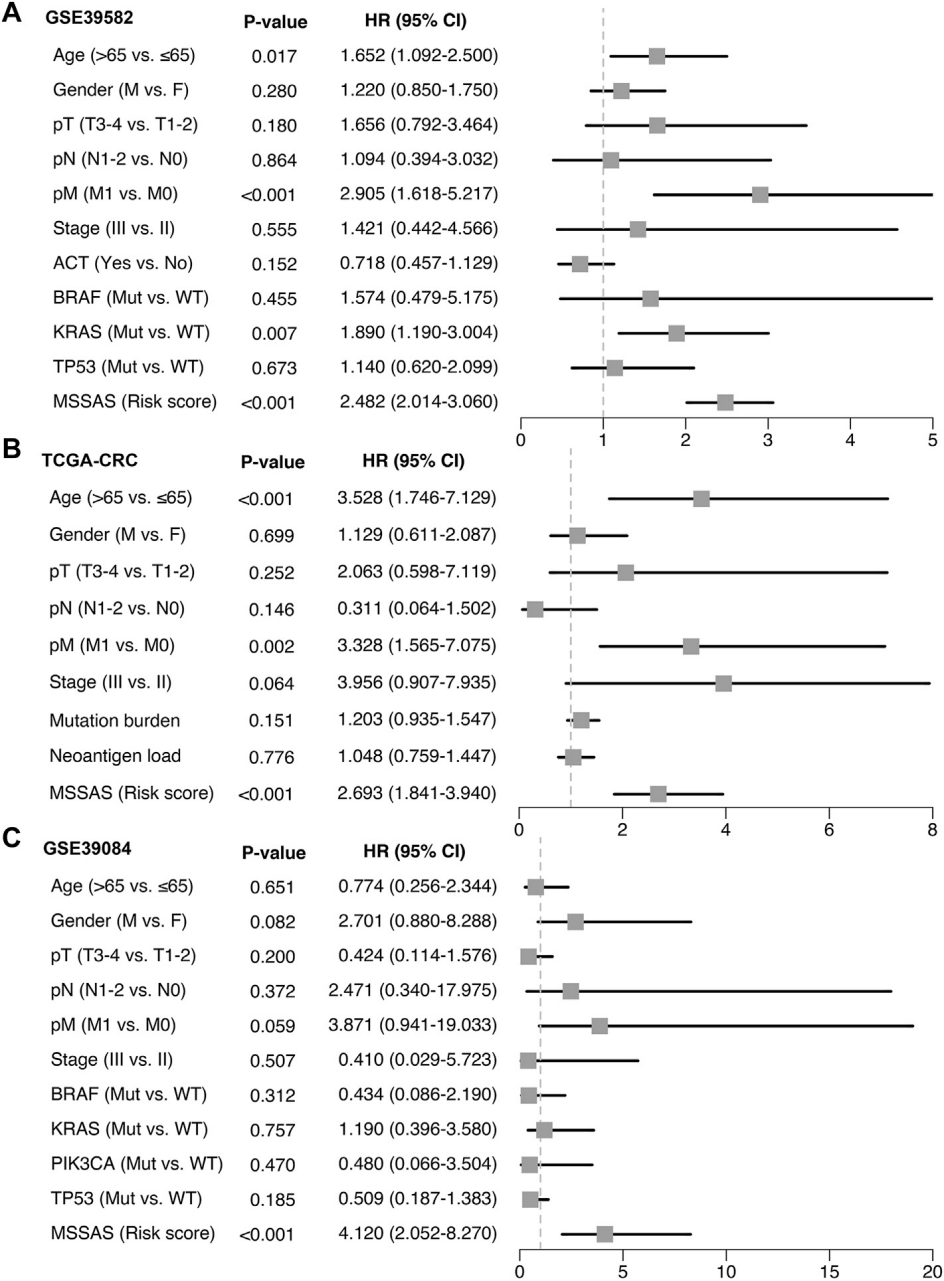
Multivariable Cox regression analysis of OS. Multivariable Cox regression analysis of MSSAS for OS in GSE39582 **(A)**, TCGA–CRC **(B)**, and GSE39084 **(C)**.

**FIGURE 5:**
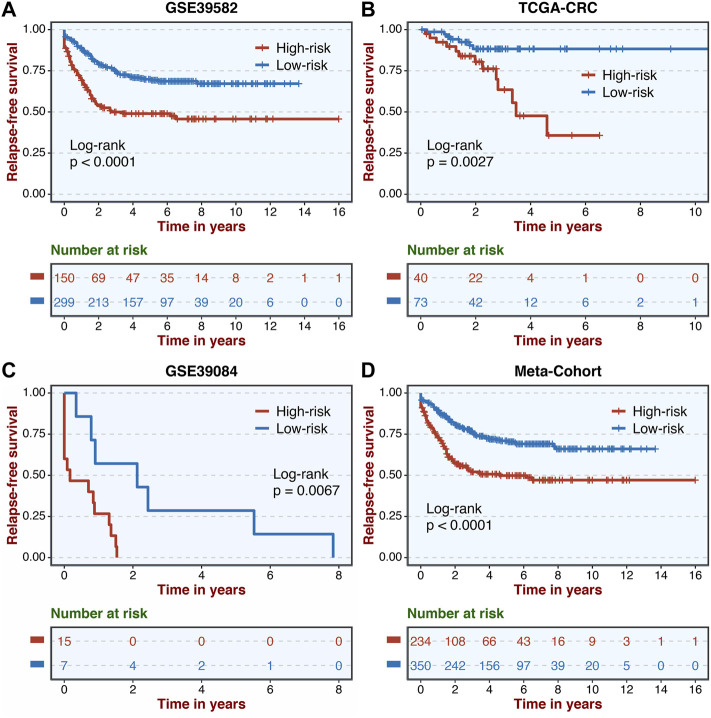
Kaplan–Meier analysis of RFS according to MSSAS. Kaplan–Meier curves of RFS according to the MSSAS in GSE39582 **(A)**, TCGA–CRC **(B)**, GSE39084 **(C)**, and Meta-Cohort **(D)**.

**FIGURE 6:**
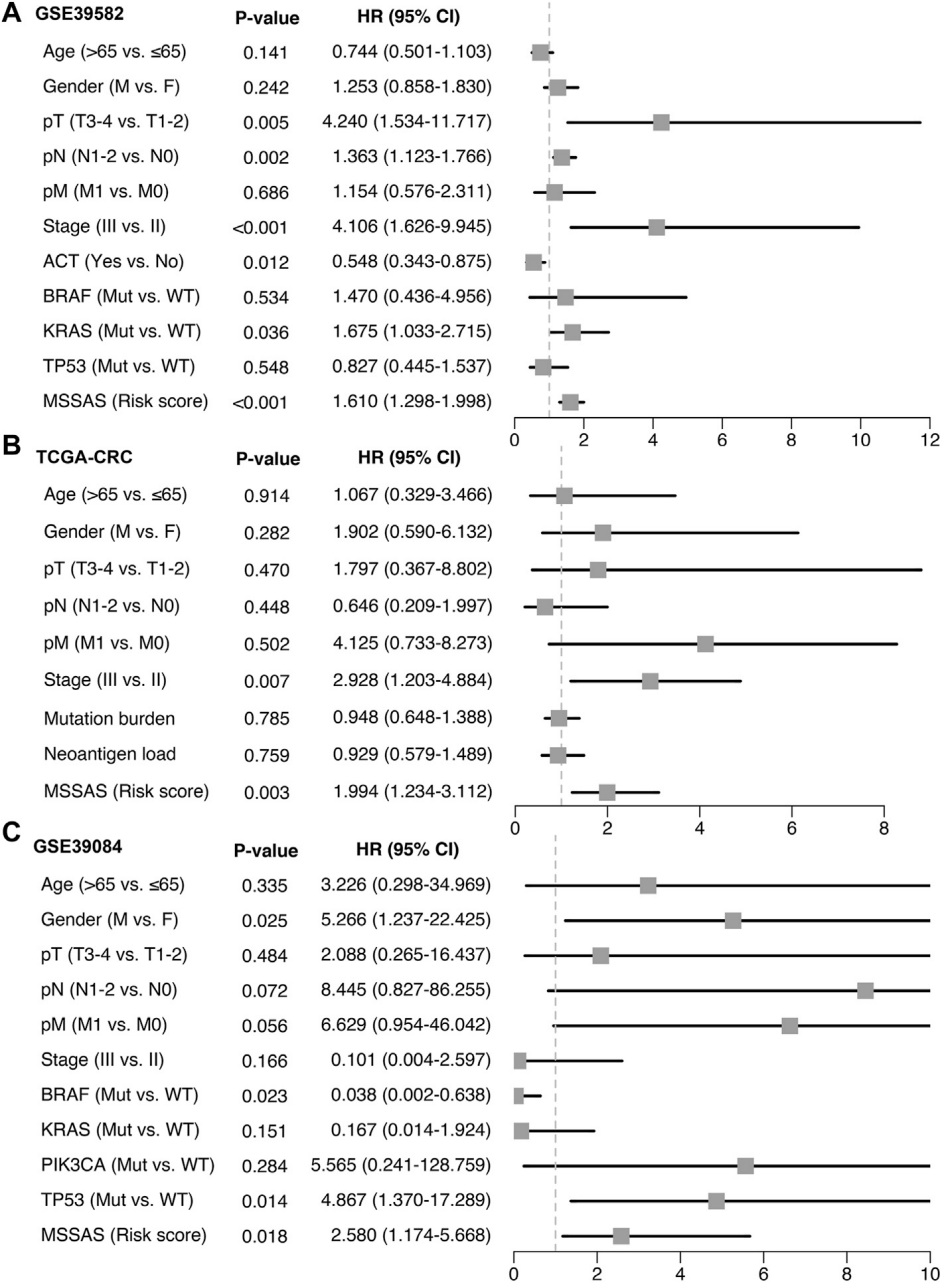
Multivariable Cox regression analysis of RFS. Multivariable Cox regression analysis of MSSAS for RFS in GSE39582 **(A)**, TCGA–CRC **(B)**, and GSE39084 **(C)**.

A total of 307 overlapped genes were obtained with differential analyses for these three cohorts. After univariate Cox regression, 11 genes in the three cohorts were defined as stable MSS-related prognostic genes (Supplementary Table S3). Subsequently, LASSO Cox regression was performed to identify eight key genes, and these genes were subjected to stepwise Cox regression (Figures 2A,B). Ultimately, the MSSAS composed of six genes was developed (Figure 2C). A risk score was established according to the expression of the six genes weighted by their regression coefficients in a penalized Cox model as follows: risk score = −0.189*Exp (*ITGB8-AS1*) −0.270*Exp (*EPHB2*) −0.319*Exp (*ATOH1*) −0.196*Exp (*HACL1*) +0.237*Exp (*Lnc-ZFAT-1*) +0.311*Exp (*ERICH3*) (Figure 2C). The risk scores of all patients with non–MSI-H/pMMR CRC were calculated from this formula. The overall survival (OS) was significantly worse in the high-risk group across GSE39582 [HR: 2.490 (2.126–2.917); log-rank test, *p* < 0.0001; Figure 3A], TCGA-CRC [HR: 2.69 (2.116–3.425); log-rank test, *p* < 0.0001; Figure 3B], GSE39084 [HR: 2.930 (1.864–4.604); log-rank test, *p* < 0.0001; Figure 3C], and Meta-Cohort [HR: 2.584 (2.282–2.925); log-rank test, *p* < 0.0001; Figure 3D] than in the low-risk group. After controlling for gender; age; T stage, N stage, M stage, and AJCC stage; adjuvant chemotherapy (ACT); tumor mutation burden (TMB); neoantigen; and *TP53*, *KRAS*, *BRAF*, and *PIK3CA* mutations, MSSAS remained statistically significant for predicting the OS in the three cohorts, which suggested that MSSAS was an independent risk factor for the OS (Figures 4A–C). Furthermore, we next explored the clinical significance of MSSAS in assessing the relapse-free survival (RFS). Similarly, the high-risk group also has dramatically dismal RFS in GSE39582 [HR: 1.674 (1.468–1.909); log-rank test, *p* < 0.0001; Figure 5A], TCGA-CRC [HR: 1.796 (1.037–3.110); log-rank test, *p* = 0.0027; Figure 5B], GSE39084 [HR: 1.892 (1.069–3.349); log-rank test, *p* = 0.0067; Figure 5C], and Meta-Cohort [HR: 1.697 (1.550–1.917); log-rank test, *p* < 0.0001; Figure 5D]. After the adjustments of available clinical variables, MSSAS remained statistically significant, which revealed that MSSAS was also an independent risk factor for RFS in non–MSI-H/pMMR CRC (Figures 6A–C).

### Signature Predictive Capability

**FIGURE 7:**
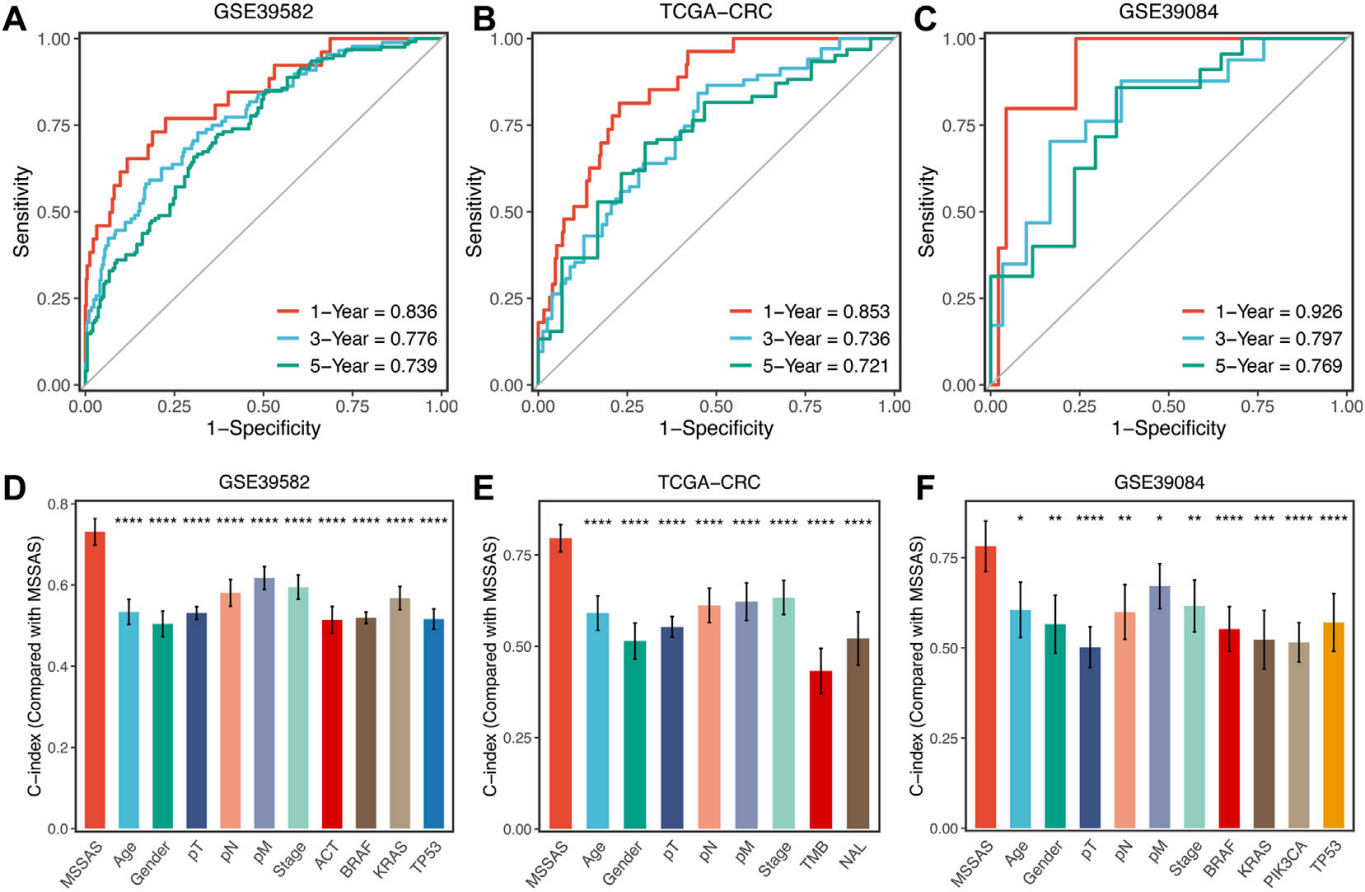
Evaluation of MSSAS in three cohorts. Time-dependent ROC analysis for predicting OS at 1, 3, and 5 years in GSE39582 **(A)**, TCGA–CRC **(B)**, and GSE39084 **(C)**. The performance of MSSAS was compared with common clinical and molecular variables in predicting prognosis across GSE39582 **(D)**, TCGA-CRC **(E)**, and GSE39084 **(F)**.

Subsequently, we evaluated the predictive capability of MSSAS in patients with non–MSI-H/pMMR CRC. The ROC analyses displayed that the areas under the curve of 1, 3, and 5 years were 0.836, 0.776, and 0.739 for GSE39582 (Figure 7A); 0.853, 0.736, and 0.721 for TCGA-CRC (Figure 7B); and 0.926, 0.797, and 0.769 for GSE39084 (Figure 7C), respectively. The C-indexes were 0.731 (0.689–0.773) in GSE39582, 0.796 (0.747–0.844) in TCGA-CRC, and 0.782 (0.691–0.873) in GSE39084, separately. These results revealed the high precision and value of MSSAS in forecasting the prognosis of non–MSI-H/pMMR CRC patients within 5 years. Moreover, MSSAS showed better performance than gender; age; T, N, M, and AJCC stages; ACT; TMB; neoantigen; and *TP53*, *KRAS*, *BRAF*, and *PIK3CA* mutations (Figures 7D–F), which indicated that MSSAS might be a promising surrogate in clinical settings.

### Validation of the Microsatellite Stable–Associated Signature in Our Clinical Cohort

**FIGURE 8:**
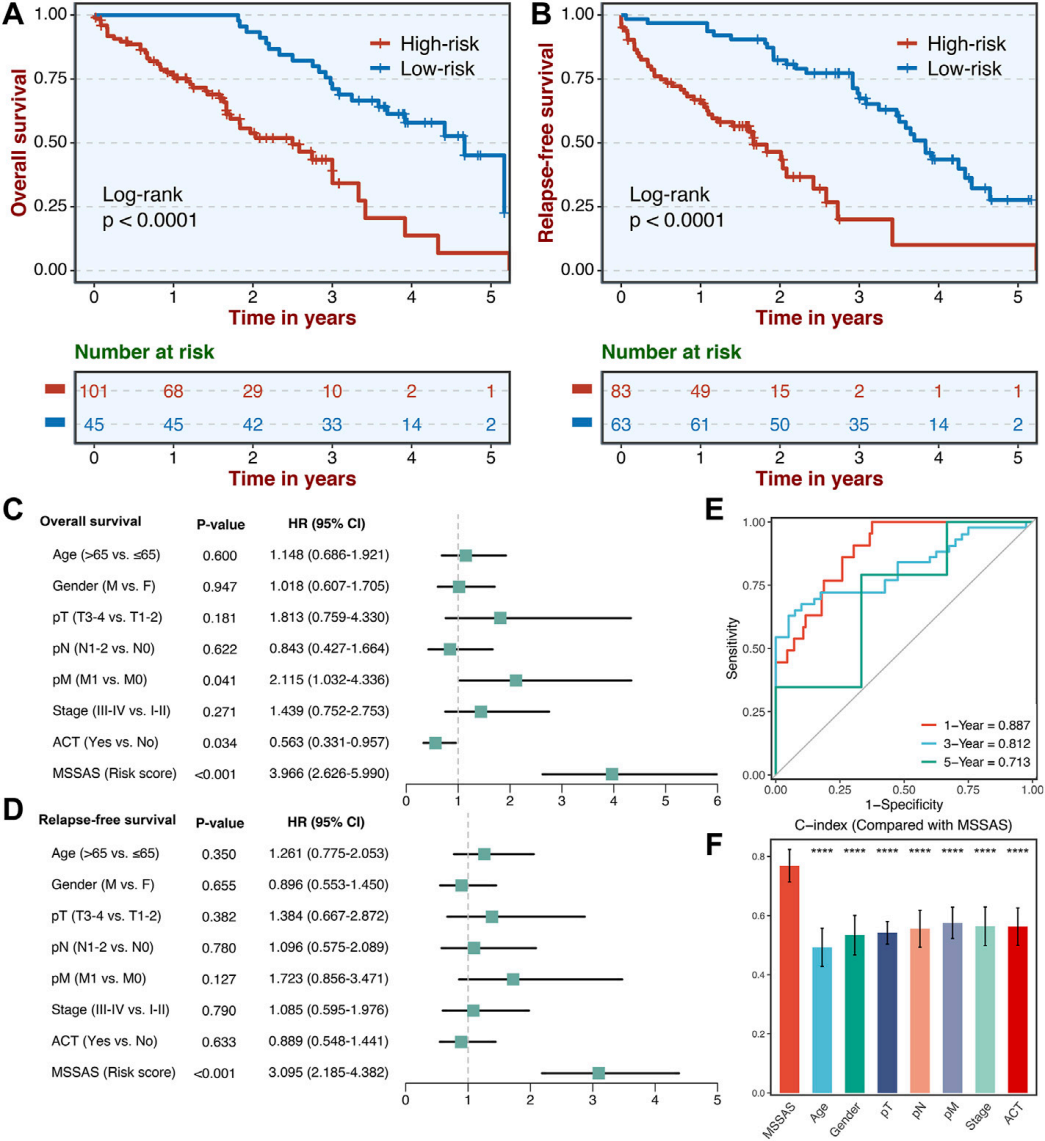
Validation of MSSAS in our clinical cohort. Kaplan–Meier curves of OS **(A)** and RFS **(B)** according to the MSSAS. Multivariate Cox regression analysis of MSSAS for OS **(C)** and RFS **(D)**. **(E)** Time-dependent ROC analysis for predicting OS at 1, 3, and 5 years. **(F)**. The performance of MSSAS was compared with common clinical and molecular variables in predicting prognosis.

A total of 146 non–MSI-H CRC tissues were collected. The qRT-PCR assays were conducted to confirm the clinical translations of this six-genes signature. Consistently, patients in the high-risk group presented much worse OS and RFS than those in the low-risk group (Figures 8A,B). Also, multivariate Cox regression analysis indicated that MSSAS remained remarkably significant for the OS [HR: 3.966 (2.626–5.990); log-rank test, *p* < 0.001; Figure 8C] and RFS [HR: 3.095 (2.185–4.382); log-rank test, *p* < 0.001; Figure 8D] after adjusting for other clinical traits in non–MSI-H/pMMR CRC. The areas under the curve were 0.887, 0.812, and 0.713 at 1, 3, and 5 years, respectively (Figure 8E), and the C-index was also much more significant than other traditional factors (Figure 8F). These results indicate that MSSAS displayed a robust and powerful performance in assessing the prognosis of non–MSI-H/pMMR CRC patients.

## Discussion

CRC is a highly heterogeneous malignancy with complicated pathological processes and mechanisms (Picard et al., 2020; Liu et al., 2021d). 5-Fluorouracil chemotherapy alone or chemotherapy in combination with radiotherapy is the standard treatment for advanced CRC (Maiuthed et al., 2018; Dekker et al., 2019). Compared with MSI-H/dMMR CRC, non–MSI-H/pMMR CRC generally has a higher response rate to 5-fluorouracil–based chemotherapy (Webber et al., 2015). Unfortunately, the prognosis and recurrence rate of non–MSI-H/pMMR CRC patients are significantly worse (Vilar and Gruber, 2010). In recent years, immunotherapy has caused a great sensation due to its significant effect on the treatments of solid tumors (Liu et al., 2021a). Immunotherapy has improved the prognosis for patients with MSI-H/dMMR CRC indeed, but it has not been effective in non–MSI-H/pMMR CRC, which accounts for 95% of metastatic CRC (Le et al., 2015; Eng et al., 2019). Therefore, in view of the worse prognosis, higher recurrence rate, and limitations of immunotherapy in non–MSI-H/pMMR CRC, evaluating the risk of non–MSI-H/pMMR CRC to select the optimized clinical intervention is necessary.

Benefiting from the emergence of bioinformatics and artificial intelligence, the use of machine learning algorithms can screen out several core indicators that are important for predicting prognosis from a large scale of gene sets (Tibshirani, 1997). This actually conforms to a biological scale-free network dominated by several hub nodes (Zhang and Horvath, 2005). Hence, a new signature to assess the prognosis and recurrence risk of non–MSI-H/pMMR CRC was developed for the first time. The stable and reproducible results of MSSAS on multiple data sets and our qRT-PCR experimental data suggest that it not only is a stable and highly reliable signature but also has outstanding specificity and sensitivity. In addition, our model has a high value of clinical application given that qRT-PCR is simple and inexpensive to perform.

In our work, a prognosis and the relapse signature consisting of six genes were constructed, including *ITGB8-AS1*, *EPHB2*, *ATOH1*, *HACL1*, *ERICH3*, and *Lnc-ZFAT-1*. Studies have confirmed that most of these genes are associated with the development of tumors. For instance, *ITGB8-AS1* has been shown to be a biomarker for the growth and migration of CRC (Lin et al., 2021). The inactivation of *EPHB2* accelerates the occurrence of CRC. Besides, *EPHB2* mutations are closely associated with tumor suppression in gastric cancer and involved in the prognosis and metastasis of other tumors such as Ewing sarcoma (Davalos et al., 2007; Keskin et al., 2021). *ATOH1* is a target of the *JNK1* and *MUC2* pathways and closely associated with the proliferation, invasion, and metastasis of CRC cells (Shen et al., 2018). *HACL1* is closely related to small-cell lung cancer, and *ERICH3* may be a key biomarker in nasopharyngeal carcinoma (Owonikoko et al., 2014; Zhang et al., 2019). The relationship of *Lnc-ZFAT-1* with cancer has not been reported in studies and needs to be further explored. Based on these six key genes, the MSSAS signature was finally established, which has been excellently displayed in predicting the prognosis of non–MSI-H/pMMR CRC patients. Multiple Cox regression analysis demonstrated that MSSAS was an independent feature after adjusting for other traditional clinical characteristics. What is more important, in three external data sets, MSSAS illustrated high calibration and discrimination in estimating the prognosis of non–MSI-H/pMMR CRC at 1, 3, and 5 years. In order to test the extrapolation ability of our signature, we performed qRT-PCR experiments on 146 MSI-H FFPE CRC tissues, and then validated the model according to its results, which showed that our signature is reliable and has high practicability in different cohorts. Besides, as we have previously demonstrated, patients with a score showed poor prognosis and RFS.

As far as we know, no one has yet developed prognostically relevant gene markers for non–MSI-H/pMMR CRC patients, and our signature fills the gap in this field. Prior to us, investigators have developed a number of biomolecular markers to predict the clinical outcomes of CRC patients (Jorissen et al., 2009; Salazar et al., 2011; Li et al., 2020). Compared with these studies, our signature has several advantages and innovations: 1) fewer genes have been included in the signature, making MSSAS easier to implement; 2) a variety of statistical algorithms were performed to estimate the calibration and discrimination of our signature. This maintained robust and powerful results at 1, 3, and 5 years; 3) external validation was performed using qRT-PCR experiments to confirm the accuracy and extrapolation ability of MSSAS. Despite the great potential clinical application value of MSSAS signature, it also has some limitations, for example, a prospective multicenter cohort is still necessary to further verify its performance.

In summary, using a systematic and comprehensive biomarker discovery and validation approach, we developed and experimentally validated a six-genes signature with a stable and powerful performance in evaluating the prognosis of non–MSI-H/pMMR CRC. It has demonstrated that our MSSAS signature might be a promising biomarker to facilitate the clinical management for non–MSI-H/pMMR CRC patients.

## Data Availability

The original contributions presented in the study are included in the article/Supplementary Material, and further inquiries can be directed to the corresponding author.

## References

[B1] Biller L. H. Schrag D. (2021). Diagnosis and Treatment of Metastatic Colorectal Cancer. JAMA 325 (7), 669–685. 10.1001/jama.2021.0106 33591350

[B2] Carethers J. M. Doubeni C. A. (2020). Causes of Socioeconomic Disparities in Colorectal Cancer and Intervention Framework and Strategies. Gastroenterology 158 (2), 354–367. 10.1053/j.gastro.2019.10.029 31682851 PMC6957741

[B3] Davalos V. Dopeso H. Velho S. Ferreira A. M. Cirnes L. Díaz-Chico N. (2007). High EPHB2 Mutation Rate in Gastric but Not Endometrial Tumors with Microsatellite Instability. Oncogene 26 (2), 308–311. 10.1038/sj.onc.1209780 16819508

[B4] Dekker E. Tanis P. J. Vleugels J. L. A. Kasi P. M. Wallace M. B. (2019). Colorectal Cancer. The Lancet 394 (10207), 1467–1480. 10.1016/S0140-6736(19)32319-0 31631858

[B5] Endo E. Okayama H. Saito K. Nakajima S. Yamada L. Ujiie D. (2020). A TGFβ-dependent Stromal Subset Underlies Immune Checkpoint Inhibitor Efficacy in DNA Mismatch Repair-Deficient/Microsatellite Instability-High Colorectal Cancer. Mol. Cancer Res. 18 (9), 1402–1413. 10.1158/1541-7786.MCR-20-0308 32493700

[B6] Eng C. Kim T. W. Bendell J. Argilés G. Tebbutt N. C. Di Bartolomeo M. (2019). Atezolizumab with or without Cobimetinib versus Regorafenib in Previously Treated Metastatic Colorectal Cancer (IMblaze370): a Multicentre, Open-Label, Phase 3, Randomised, Controlled Trial. Lancet Oncol. 20 (6), 849–861. 10.1016/S1470-2045(19)30027-0 31003911

[B7] Ganesh K. Stadler Z. K. Cercek A. Mendelsohn R. B. Shia J. Segal N. H. (2019). Immunotherapy in Colorectal Cancer: Rationale, Challenges and Potential. Nat. Rev. Gastroenterol. Hepatol. 16 (6), 361–375. 10.1038/s41575-019-0126-x 30886395 PMC7295073

[B8] Jorissen R. N. Gibbs P. Christie M. Prakash S. Lipton L. Desai J. (2009). Metastasis-Associated Gene Expression Changes Predict Poor Outcomes in Patients with Dukes Stage B and C Colorectal Cancer. Clin. Cancer Res. 15 (24), 7642–7651. 10.1158/1078-0432.ccr-09-1431 19996206 PMC2920750

[B9] Kather J. N. Halama N. Jaeger D. (2018). Genomics and Emerging Biomarkers for Immunotherapy of Colorectal Cancer. Semin. Cancer Biol. 52 (Pt 2), 189–197. 10.1016/j.semcancer.2018.02.010 29501787

[B10] Keskin T. Rucci B. Cornaz-Buros S. Martin P. Fusco C. Broye L. (2021). A Live Single-Cell Reporter Assay Links Intratumor Heterogeneity to Metastatic Proclivity in Ewing Sarcoma. Sci. Adv. 7 (27), 7. 10.1126/sciadv.abf9394 PMC11060044 34215585

[B11] Le D. T. Uram J. N. Wang H. Bartlett B. R. Kemberling H. Eyring A. D. (2015). PD-1 Blockade in Tumors with Mismatch-Repair Deficiency. N. Engl. J. Med. 372 (26), 2509–2520. 10.1056/NEJMoa1500596 26028255 PMC4481136

[B12] Li J. Zhang J. Hu H. Cai Y. Ling J. Wu Z. (2020). Gene Expression Signature to Predict Prognosis and Adjuvant Chemosensitivity of Colorectal Cancer Patients. Cmar Vol. 12, 3301–3310. 10.2147/CMAR.S243490 PMC7227814 32494194

[B13] Lin X. Zhuang S. Chen X. Du J. Zhong L. Ding J. (2021). lncRNA ITGB8-AS1 Functions as a ceRNA to Promote Colorectal Cancer Growth and Migration through Integrin-Mediated Focal Adhesion Signaling. Mol. Ther. S1525-0016, 00405–00406. 10.1016/j.ymthe.2021.08.011 PMC8821934 34371180

[B14] Liu Z. Lu T. Li J. Wang L. Xu K. Dang Q. (2021). Development and Clinical Validation of a Novel Six-Gene Signature for Accurately Predicting the Recurrence Risk of Patients with Stage II/III Colorectal Cancer. Cancer Cel Int 21 (1), 359. 10.1186/s12935-021-02070-z PMC8265123 34233675

[B15] Liu Z. Lu T. Li J. Wang L. Xu K. Dang Q. (2021). Clinical Significance and Inflammatory Landscape of aNovel Recurrence-Associated Immune Signature in Stage II/III Colorectal Cancer. Front. Immunol. 12, 702594. 10.3389/fimmu.2021.702594 34394098 PMC8358813

[B16] Liu Z. Lu T. Wang Y. Jiao D. Li Z. Wang L. (2021). Establishment and Experimental Validation of an Immune miRNA Signature for Assessing Prognosis and Immune Landscape of Patients with Colorectal Cancer. J. Cel Mol Med 25 (14), 6874–6886. 10.1111/jcmm.16696 PMC8278100 34101338

[B17] Liu Z. Zhang Y. Dang Q. Wu K. Jiao D. Li Z. (2021). Genomic Alteration Characterization in Colorectal Cancer Identifies a Prognostic and Metastasis Biomarker: FAM83A|Ido1. Front. Oncol. 11, 632430. 10.3389/fonc.2021.632430 33959500 PMC8093579

[B18] Lizardo D. Y. Kuang C. Hao S. Yu J. Huang Y. Zhang L. (2020). Immunotherapy Efficacy on Mismatch Repair-Deficient Colorectal Cancer: From Bench to Bedside. Biochim. Biophys. Acta (Bba) - Rev. Cancer 1874 (2), 188447. 10.1016/j.bbcan.2020.188447 PMC7886024 33035640

[B19] Maiuthed A. Ninsontia C. Erlenbach-Wuensch K. Ndreshkjana B. Muenzner J. Caliskan A. (2018). Cytoplasmic P21 Mediates 5-Fluorouracil Resistance by Inhibiting Pro-apoptotic Chk2. Cancers 10 (10), 373. 10.3390/cancers10100373 PMC6210175 30304835

[B20] Owonikoko T. K. Zhang G. Deng X. Rossi M. R. Switchenko J. M. Doho G. H. (2014). Poly ( ADP ) Ribose Polymerase Enzyme Inhibitor, Veliparib, Potentiates Chemotherapy and Radiation *In Vitro* and *In Vivo* in Small Cell Lung Cancer. Cancer Med. 3 (6), 1579–1594. 10.1002/cam4.317 25124282 PMC4298385

[B21] Picard E. Verschoor C. P. Ma G. W. Pawelec G. (2020). Relationships between Immune Landscapes, Genetic Subtypes and Responses to Immunotherapy in Colorectal Cancer. Front. Immunol. 11, 369. 10.3389/fimmu.2020.00369 32210966 PMC7068608

[B22] Salazar R. Roepman P. Capella G. Moreno V. Simon I. Dreezen C. (2011). Gene Expression Signature to Improve Prognosis Prediction of Stage II and III Colorectal Cancer. Jco 29 (1), 17–24. 10.1200/JCO.2010.30.1077 21098318

[B23] Shen P. Yang S. Sun H. Li G. Wu B. Ji F. (2018). SCF/c-KIT Signaling Increased Mucin2 Production by Maintaining Atoh1 Expression in Mucinous Colorectal Adenocarcinoma. Ijms 19 (5), 1541. 10.3390/ijms19051541 PMC5983812 29786668

[B24] Tibshirani R. (1997). The Lasso Method for Variable Selection in the Cox Model. Statist. Med. 16 (4), 385–395. 10.1002/(sici)1097-0258(19970228)16:4<385:aid-sim380>3.0.co;2-3 9044528

[B25] Vilar E. Gruber S. B. (2010). Microsatellite Instability in Colorectal Cancer-The Stable Evidence. Nat. Rev. Clin. Oncol. 7 (3), 153–162. 10.1038/nrclinonc.2009.237 20142816 PMC3427139

[B26] Webber E. M. Kauffman T. L. O’Connor E. Goddard K. A. (2015). Systematic Review of the Predictive Effect of MSI Status in Colorectal Cancer Patients Undergoing 5FU-Based Chemotherapy. BMC Cancer 15, 156. 10.1186/s12885-015-1093-4 25884995 PMC4376504

[B27] Zhang B. Horvath S. (2005). A General Framework for Weighted Gene Co-expression Network Analysis. Stat. Appl. Genet. Mol. Biol. 4, Article17. Article17. 10.2202/1544-6115.1128 16646834

[B28] Zhang J.-Z. Wu Z.-H. Cheng Q. (2019). Screening and Identification of Key Biomarkers in Nasopharyngeal Carcinoma. Medicine (Baltimore) 98 (48), e17997. 10.1097/MD.0000000000017997 31770211 PMC6890310

[B29] Zhang Y. Liu Z. Li X. Liu L. Wang L. Han X. (2021). Comprehensive Molecular Analyses of a Six-Gene Signature for Predicting Late Recurrence of Hepatocellular Carcinoma. Front. Oncol. 11, 732447. 10.3389/fonc.2021.732447 34568069 PMC8459683

