# Supplementary material for: Gene Expression Profile Reveals a Prognostic Signature of Non–MSI-H/pMMR Colorectal Cancer
Source: Front Cell Dev Biol. 2022 Feb 17;10:790214. doi: 10.3389/fcell.2022.790214 (PMC8891566; doi:10.3389/fcell.2022.790214)
Supplement: Supplementary file 3 [file Table1.DOCX]

**Supplementary material**

**Data processing**

The colorectal cancer data were enrolled from The Cancer Genome Atlas (TCGA) cohorts TCGA-COAD (colon adenocarcinoma) and TCGA-READ (rectum adenocarcinoma). “Level 3” transcriptome data (RNA-Seq raw read count) and clinical information were retrieved from TCGA data portal (<https://portal.gdc.cancer.gov/>). Patients from TCGA were defined as TCGA-CRC cohort. The RNA-seq raw read count from TCGA database was converted to transcripts per kilobase million (TPM). A further log-2 transformation was performed due to RNA-seq data is often heavily right-skewed in the linear scale, which is more similar with the distribution of microarray data and more comparable between samples. The mRNAs with zero reads in all samples were further excluded. GSE39582 and GSE39084, used Affymetrix human genome U133 plus 2.0 array for gene annotation. Data was normalized with robust multiarray averaging (RMA) method in the affy package. RMA was used to perform background adjustment, quantile normalization, and final summarization of oligonucleotides per transcript using the median polish algorithm. In three cohorts, we only retained CRC patients that met the following criteria: (1) Have mRNA expression data; (2) Have survival information; (3) Have microsatellite state information; (4) No preoperative chemotherapy or radiotherapy received. Ultimately, TCGA-CRC has 54 high microsatellite instability (MSI-H) CRC and 312 non-MSI-H CRC; GSE39582 has 77 deficient mismatch repair (dMMR) CRC and 459 proficient mismatch repair (pMMR) CRC; GSE39084 has 16 MSI-H CRC and 54 non-MSI-H CRC. The corresponding clinical information of three cohorts was also downloaded, and the baseline data were summarized in Table S1.

**qRT-PCR assay of 146 FFPE samples**

From January 2015 to December 2020, we collected a total of 146 frozen surgically resected CRC tissues with MSI-L/MSS at The First Affiliated Hospital of Zhengzhou University. Clinical staging of the specimens was based on NCCN (2019) guidelines. Detailed baseline data of CRC patients are displayed in Table S1. Total RNA was isolated from CRC tissues using RNAiso Plus reagent (Takara, Dalian, China) according to the manufacturer’s instructions. RNA quality was evaluated using a NanoDrop One C (Waltham, MA, USA), and RNA integrity was assessed using agarose gel electrophoresis. An aliquot of 1 µg of total RNA was reverse-transcribed into complementary DNA (cDNA) according to the manufacturer’s protocol using the miRNA reverse transcription Kit (TaKaRa BIO, Japan). All cDNA samples were prepared for qRT-PCR. This project was approved by the Ethics Committee Board of The First Affiliated Hospital of Zhengzhou University. In the qRT-PCR analysis, the enrolled 6 genes in the MSSAS signature were detected. qRT-PCR was performed using SYBR Assay I Low ROX (Eurogentec, USA) and SYBR® Green PCR Master Mix (Yeason, Shanghai, China). The expression value of the target genes was normalized to GAPDH, and then log2 transformed for subsequent analysis. The primer sequences of the included 6 genes and GAPDH were shown in Table S2.
